# An Advanced Transcriptional Response to Corticosterone After Single Prolonged Stress in Male Rats

**DOI:** 10.3389/fnbeh.2021.756903

**Published:** 2021-11-17

**Authors:** Jinlan Ding, Xinzhao Chen, Fang Han, Onno C. Meijer

**Affiliations:** ^1^Division of Endocrinology, Department of Internal Medicine, Leiden University Medical Center, Leiden University, Leiden, Netherlands; ^2^Einthoven Laboratory for Experimental Vascular Medicine, Leiden University Medical Center, Leiden University, Leiden, Netherlands; ^3^PTSD Lab, Department of Histology and Embryology, Basic Medical College, China Medical University, Shenyang, China

**Keywords:** negative feedback, corticosterone, GR sensitivity, FKBP5, hippocampus

## Abstract

Stress-related neuropsychiatric disorders are often accompanied by dysfunction of the hypothalamic-pituitary-adrenal (HPA) axis. In patients suffering from post-traumatic stress disorder (PTSD), increased sensitivity of glucocorticoid negative feedback has regularly been observed. The single prolonged stress (SPS) paradigm was developed to model increased negative feedback and other aspects of PTSD in rats. In this study, we used a setup that precluded the evaluation of negative feedback but rather served to test the hypothesis of the enhanced glucocorticoid receptor (GR) signaling in higher brain areas. We injected corticosterone or vehicle 7 days after SPS and evaluated plasma corticosterone, as well as gene expression in the dorsal hippocampus and amygdala. We observed a strikingly rapid change in the expression of established GR target genes (*t* = 30 min) only in the SPS group on exogenous corticosterone injection. Our results extend the notion of increased GR sensitivity in PTSD to include transcriptional responses in the hippocampus.

## Introduction

In physiological conditions, glucocorticoid hormone levels increase systemically in response to stress, as a consequence of the activation of the hypothalamic-pituitary-adrenal (HPA) axis (Heim and Nemeroff, 2001; Smith and Vale, 2006; Jacobson, 2014; Boero et al., 2018). Stress-related neuropsychiatric disorders are often accompanied by the dysfunction of the HPA axis. Specifically, patients suffering from post-traumatic stress disorder (PTSD) show alterations of the HPA system (Ströhle et al., 2008). Prior studies reported inconsistent data on basal cortisol levels in individuals with PTSD (Baker et al., 2005; Meewisse et al., 2007). However, the general consensus is that these patients exhibit increased sensitivity of glucocorticoid negative feedback (Kanter et al., 2001), based on, for example, the dexamethasone suppression test and the metyrapone stimulation test (Yehuda, 2005; Yehuda et al., 2009; Daskalakis et al., 2013). The glucocorticoid negative feedback is primarily mediated by the glucocorticoid receptor (GR), the anterior pituitary (outside the brain), and the hypothalamus (Laryea et al., 2015; Herman et al., 2016).

The single prolonged stress (SPS) paradigm in rats was developed to model PTSD, including enhanced negative feedback on the HPA axis (Liberzon et al., 1997). However, GR is expressed widely in the brain and regulates the transcription of gene networks necessary for adaption to stressors (Datson et al., 2001). In fact, the changes in expression and the subcellular distribution of GR (and of the related mineralocorticoid receptor (MR)) have previously been found in the hippocampus, amygdala, and medial prefrontal cortex (Souza et al., 2017). Recent evidence suggests that hippocampal GR signaling may also be affected in a different animal model for PTSD (Danan et al., 2021). However, to our knowledge, no study has directly tested GR functionality by the evaluation of corticosterone-induced changes in gene expression in SPS rats. In this study, we tested the hypothesis that SPS affects the overall GR responsiveness in the brains of male rats. Our setup did not allow the evaluation of negative feedback sensitivity under the specific experimental conditions. However, we found that the mRNA induction of established GR target genes in the hippocampus and amygdala occurred as early as 30 min after corticosterone injection in SPS rats only.

## Materials and Methods

### Animals

Adult male Wistar rats (200–220 g, 7 weeks old) were paired-housed on a 12-h light/dark cycle and controlled conditions of temperature (light on at 7:00–19:00 at 22 ± 1°C) with standard rat diet and *ad libitum* access to water. A total of 68 animals were used in this study (32 to make 4 experimental groups of *n* = 8 for plasma collection at 3 h after injection and 36 to make 6 experimental groups of *n* = 6 for the gene response experiment at 0.5 h after injection). Animal procedures were approved by the China Medical University Animal Care and were performed in accordance with the Chinese National Guidelines on Animal Care.

### Drugs

Rats were injected intraperitoneally with vehicle (5% ethanol in phosphate-buffered saline (PBS)) or corticosterone (3 mg/kg). Corticosterone (Sigma, St. Louis, MO, United States) was dissolved in 100% ethanol, diluted to a final 5% ethanol solution in normal PBS, and injected in a volume of 5 ml/kg. The doses of corticosterone we used led to plasma corticosterone concentrations in the range of those observed after stress (Cohen et al., 2006; Kaouane et al., 2012).

### Experimental Design

Rats were allowed to adapt for 1 week prior to initiate the experimental protocols. All experimental procedures were started at 9:00. On day 0, rats were subjected to the SPS paradigm. The single session of prolonged stress was performed as previously described (Liberzon and Young, 1997). SPS consisted of restraint for 2 h in an acrylic animal holder followed immediately by a forced swim for 20 min in 24°C freshwater (water depth: 40 cm). Animals were given 15 min to recuperate and were then exposed to the ether vapor until the loss of consciousness. The animals were then returned to their home cage and left undisturbed for 7 days (to allow the behavioral phenotypes relevant to the PTSD symptomatology to develop). Control animals remained in their home cage with no handling and were injected and sacrificed at the same time with the stressed groups.

**FIGURE 1:**
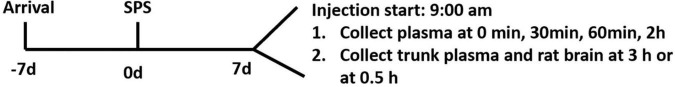
Schematic diagram of the study. Of note, 1 week after arrival in the facility, rats were exposed to single prolonged stress (SPS; day 0); 7 days after SPS, rats were injected with corticosterone or vehicle. In experiment 1, plasma was collected *via* a tail cut at 0, 30, 60 min, and 2 h. Rats were sacrificed at 3 h (experiment 1) or 0.5 h (experiment 2) after injection.

On day 7, animals were injected with corticosterone or vehicle according to the body weight, leading to control-vehicle (CV), control-corticosterone (CC), SPS-vehicle (SV), and SPS-corticosterone (SC) groups. In one experiment, blood was collected from the caudal vein at 0, 30, 60 min, and 2 h, and all rats were sacrificed to collect brains at 3 h after injection. In a second experiment, we sacrificed the rats at 0.5 h after injection, and the trunk blood and brains were collected. In the second experiment, we also included non-injected rats. The design of the experiment is outlined in Figure 1.

### General Body Parameters of the Second Experiment

Body weight was determined using the weighing scale on day 0, 3, and 7 after SPS. Baseline body weight at day 0 was 249 ± 17 g on average. We expressed the gain in body weight relative to the start of the SPS exposure. Food and water intake were recorded from day 0 to day 7.

### ELISA Analysis for Corticosterone

The blood samples were collected in heparinized capillaries and centrifuged at 12,000 rpm for 5 min to remove blood cells and obtain plasma, and then, those were stored at −80°C till the measurements were carried out. The plasma concentration of corticosterone was quantified using ELISA (AC-15F1, Immunodiagnostic Systems, United Kingdom) according to the manual of the manufacturer.

### Determination of Changes in mRNA Levels for Candidate Genes in the Dorsal Hippocampus and Amygdala

Following the sacrifice, brains were immediately removed and frozen on dry ice. Notably, 80-μm sections were cut on a cryostat, and the dorsal hippocampus from Bregma −2.40 mm to Bregma −4.36 mm, according to the study by Paxinos and Watson (2007), and the amygdala (i.e., the central amygdala and the basolateral complex and part of the medial nucleus) from Bregma −2.16 mm to Bregma −3.36 mm (Paxinos and Watson, 2007) were punched out using a 1-mm sample corer (Fine Science Tools, Foster City, CA, United States). Total mRNA was isolated, and the concentrations were determined using a NanoDrop 2000 (Thermo Fisher Scientific, Pittsburgh, PA, United States). cDNA synthesis and qPCR were performed as per the instructions of the manufacturer. Data were normalized to glyceraldehyde-3-phosphate (GAPDH) mRNA and expressed as the relative fold change calculated using the 2^–ΔΔ*Ct*^ method. Tested genes and their primers are described in Table 1.

**TABLE 1:** Primer sequences for qPCR.

**Gene**	**Forward primer (5′-3′)**	**Reverse primer (5′-3′)**
GAPDH	ACGGCAAGTTCAACGGCACAG	AAGACGCCAGTAGACTCCACGACA
FKBP5	AAGCATTGAGCAAGAAGGCAGTA	GAGGAGGGCCGAGTTCATTAG
Irs2	GGAAGTCTGTTCGGGTGTGT	AGTGCAGGTTCCTCGTCAAC
Ntf3	CAAGTCCTCAGCCATTGACA	CTGGCCTGGCTTCTTTACAC
Drd1a	AGATCCATCGAGTCCCCTCT	TGTTGCAACTGCTTCCAAAG

### Statistical Analysis

Data are expressed as mean ± SEM. The statistical analysis was conducted with the unpaired Student’s *t*-test or two-way ANOVA followed by the *post hoc* Tukey’s multiple comparison test (as appropriate) using GraphPad Prism 8.0 software. The results were considered statistically significant at *p* < 0.05.

## Results

### Food/Water Intake and Body Weight Parameters

**FIGURE 2:**
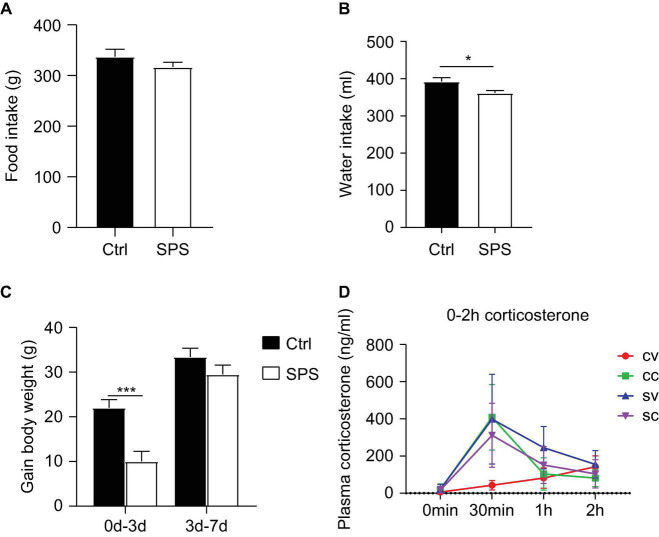
Effects of SPS on food and water intake, body parameters, and corticosterone levels after injections. **(A)** Food consumption, **(B)** water consumption, **(C)** gain in body weight, and **(D)** corticosterone levels at 0 min, 0.5, 1, and 2 h after injection. CV, control+vehicle group; CC, control+CORT group; SV, SPS+vehicle group; SC, SPS+CORT group. *p < 0.05, ***p < 0.001.

Data on food/water intake and body weights were consistent in both experiments. In this study, we have only shown the data of the second experiment. Total food intake in the week after the SPS procedure did not significantly differ from the control group (Figure 2A). However, the water intake of the SPS group was significantly reduced compared to the control group (*t* = 2.416, *p* < 0.05, Figure 2B). The control rats gained more body weight than the SPS group during the first 3 days after SPS (*t* = 4.097, *p* < 0.05). During the last 4 days, the body weight gain did not differ between the groups (Figure 2C).

### Hypothalamic-Pituitary-Adrenal Axis Response in Single Prolonged Stress-Control Rats

Plasma corticosterone (Figure 2D) levels were measured at different time points after injections in the first experiment to evaluate the response to vehicle injection. The corticosterone levels showed an interaction effect and a trend toward a SPS main effect at 30 min after injection [interaction: *F*_(1, 11)_ = 12.84, *p* < 0.05, stress: *p* = 0.078], as well as an SPS effect at 60 min after injection [stress: *F*_(1, 22)_ = 11.48, *p* < 0.01]. As expected, exogenous corticosterone injection led to similarly increased concentrations that returned to baseline after 60 min. The lack of an ANOVA corticosterone injection main effect (CORT: *p* > 0.05) *per se* could be attributed to a strong increase of corticosterone levels in the vehicle-injected SPS rats at the 30-min time point. The corticosterone levels were still elevated 60 min after vehicle injection at 60 min in the SPS group. The high levels of corticosterone in vehicle-treated SPS rats indicated enhanced stress reactivity in these animals but precluded comparing the transcriptional response to corticosterone, for the lack of a low-corticosterone SPS group.

### Gene Expression Effects of Corticosterone Half an Hour After Injection in the Dorsal Hippocampus and in the Amygdala

**FIGURE 3:**
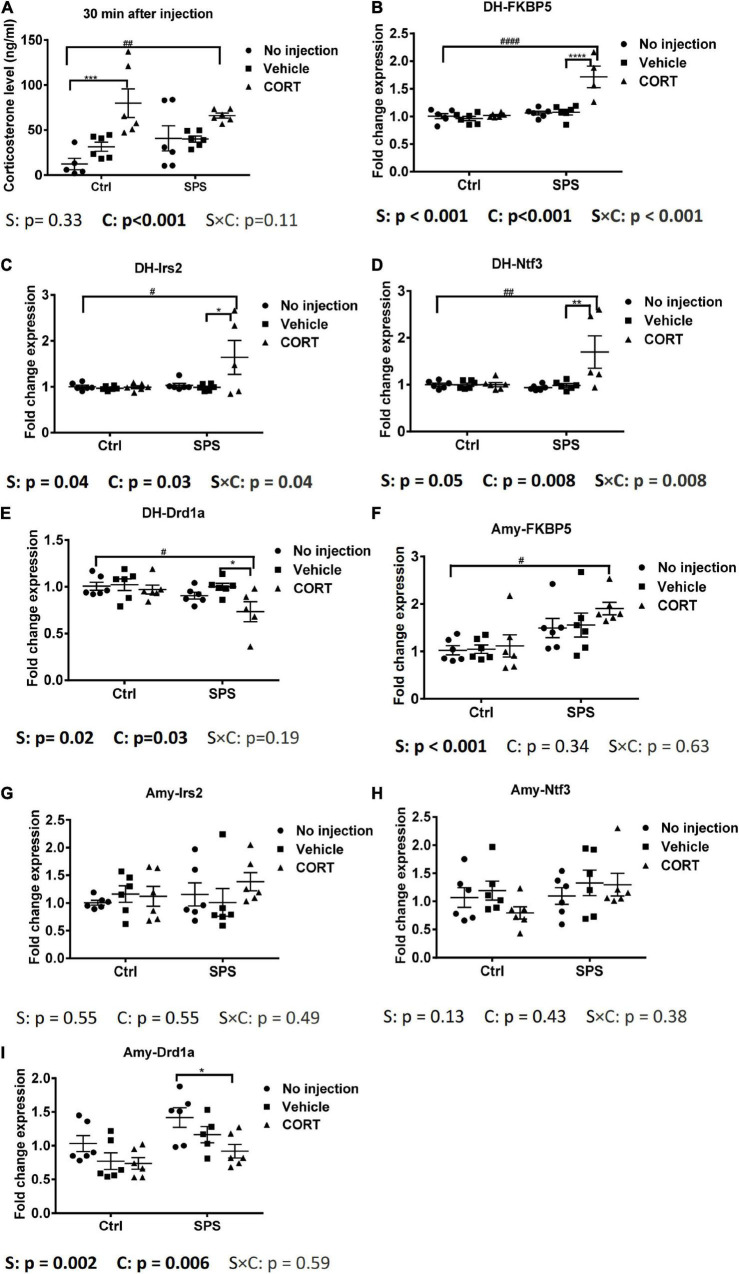
Plasma corticosterone levels and gene expression in the dorsal hippocampus and in the amygdala at 0.5 h after injection. **(A)** Corticosterone levels, **(B)** FKBP5 mRNA expression in the dorsal hippocampus, **(C)** Irs2 mRNA expression in the dorsal hippocampus, **(D)** Ntf3 mRNA expression in the dorsal hippocampus, **(E)** Drd1a mRNA expression in the dorsal hippocampus, **(F)** FKBP5 mRNA expression in the amygdala, **(G)** Irs2 mRNA expression in the amygdala, **(H)** Ntf3 mRNA expression in the amygdala, and **(I)** Drd1a mRNA expression in the amygdala. The data are expressed as mean ± SEM. Statistical significance was determined by two-way ANOVA followed by the *post hoc* Tukey’s test. #: differences between control and SPS groups; *: differences within the control or SPS groups. *p* < 0.05, ^##^*p* < 0.01, ^####^*p* < 0.001, */^#^*p* < 0.05, **/^##^*p* < 0.01, ***p < 0.001, ****/^####^*p* < 0.001.

The elevated corticosterone in SV rats in our first experiment could have been caused by the injection, the tail blood sampling, or both. To control for the acute effects of the injection itself, in our next experiment, we included SPS and control groups that did not receive an injection and compared the corticosterone levels with animals half an hour after an injection of vehicle or corticosterone. We did not apply tail cuts in these rats. The corticosterone results showed a significant exogenous CORT main effect [*F*_(2,29)_ = 13.16, *p* < 0.001, Figure 3A]. *Post hoc* test confirmed a significant increase in plasma corticosterone only in the CORT-injected control and SPS animals, relative to untreated and vehicle-treated controls.

Since the corticosterone levels were strongly induced only after the injection of the hormone, we decided to evaluate the mRNA responses in the brains of the animals in this experiment. Strikingly, in the dorsal hippocampus, the FKBP5 mRNA showed an interaction and two main effects between stress and exogenous corticosterone [interaction: *F*_(2,28)_ = 13.3, *p* < 0.001; stress: *F*_(1,28)_ = 28.72, *p* < 0.001; CORT: *F*_(2,28)_ = 16.42, *p* < 0.001, Figure 3B]. The *post hoc* analysis showed that FKBP5 mRNA levels were increased only in SPS rats after the corticosterone injection. We also evaluated the expression of additional target genes, based on some robust corticosterone-induced target genes as identified in a previous study (Datson et al., 2013). The Irs2 mRNA expression was similar to the FKBP5 mRNA expression. It showed a significant interaction and two main effects for stress and exogenous corticosterone [interaction: *F*_(2,29)_ = 3.692, *p* < 0.05; stress: *F*_(1,29)_ = 4.71, *p* < 0.05; CORT: *F*_(2,29)_ = 3.879, *p* < 0.05, Figure 3C]. In the *post hoc* comparison, the upregulation of Irs2 mRNA expression after corticosterone occurred only in SPS rats. Ntf3 mRNA levels showed a very similar pattern. The two-way ANOVA showed the effect of exogenous corticosterone and an interaction between stress and CORT [CORT: *F*_(2, 29)_ = 5.772, *p* < 0.01; interaction: *F*_(2, 29)_ = 5.697, *p* < 0.01, Figure 3D]. *Post hoc* tests showed that the Ntf3 mRNA expression was only upregulated by corticosterone in SPS rats. We selected Drd1a as a downregulated gene, which was earlier found to be downregulated irrespective of stress history (Datson et al., 2013; Zalachoras et al., 2013). For this mRNA, there were significant main effects for stress and CORT [stress: *F*_(1, 29)_ = 6.555, *p* < 0.05; CORT: *F*_(2, 29)_ = 3.898, *p* < 0.05, Figure 3E]. The *post hoc* test revealed that Drd1a mRNA levels were suppressed after exogenous corticosterone but only in SPS rats. Thus, in the hippocampus, these 4 genes responded to corticosterone after 30 min in the SPS rats only.

In the amygdala, FKBP5 mRNA levels showed a main effect of stress [stress: *F*_(1, 30)_ = 16.11, *p* < 0.001, Figure 3F]. The *post hoc* tests showed a higher mRNA level of the SPS-CORT group compared to the control without injection. There was no significant upregulation after corticosterone within the stress or control groups. There was no difference in each group for Irs2 and Ntf3 mRNA expression (Figures 3G,H). Drd1a mRNA showed main effects of stress and CORT [stress: *F*_(1, 29)_ = 11.12, *p* < 0.01; CORT: *F*_(2, 29)_ = 6.058, *p* < 0.01, Figure 3I]. In pairwise comparisons, Drd1a in the SPS with CORT injection was lower than that in the SPS without injection. In sum, in the amygdala, most genes that were identified previously as corticosterone targets in the hippocampus did not differ between groups, but for those genes that were responsive to corticosterone, the effect was observed only in rats that had undergone SPS.

## Discussion

In this study, we administered exogenous CORT to evaluate the GR sensitivity in the hippocampus and amygdala 1 week after the SPS procedure. Our rationale was the documented feedback sensitivity of the HPA axis in this model (Liberzon and Young, 1997) and the likely importance of enhanced GR sensitivity in the limbic brain regions (de Kloet et al., 2005). We found that the experimental procedure of injection and repeated blood sampling *via* the tail led to a pronounced adrenocortical activation in SPS rats, which precluded a properly controlled evaluation of GR target gene expression after 3 h. In contrast, 30 min after a vehicle injection alone, SPS rats did not show a corticosterone elevation. We then observed a striking mRNA response of up- and downregulated GR target genes in the corticosterone-treated animals, at this early time point in SPS rats. Our data suggest the enhanced stress responsiveness after SPS to moderate but not mild stressors and a sensitization of brain GR signaling that extends beyond direct negative feedback regulation.

An enhanced GR activity in models of traumatic stressors has mainly been observed for negative feedback changes. This is a complex phenomenon in itself, with both non-genomic and genomic effects of primarily GR (Dallman et al., 1989; Lightman, 2008). It involves GR activation in the pituitary (i.e., the primary target of dexamethasone) and in the brain. The responsible brain GRs reside primarily in the hypothalamic paraventricular nucleus (Dallman et al., 1989; Lightman, 2008; Schmidt et al., 2009; Tasker and Herman, 2011) and secondarily in higher brain centers project to the hypothalamus (Herman et al., 2020). In higher brain centers, GR acts in concert with MRs (de Kloet, 2003; de Kloet et al., 2008). Our understanding of the nature of enhanced feedback has remained limited, although, in patients, both pituitary and central GRs have been implicated (Yehuda et al., 2006; Cooper et al., 2017), and probing MR functionality suggested no differences (Otte et al., 2006).

Our data do not allow further insights into negative feedback strength *per se*, because SPS rats reacted strongly to the initial protocol of injection followed by the tail blood sampling. In control rats, this method may be used as a mild, essentially stress-free way of collecting blood (Fluttert et al., 2000). The enhanced stress reactivity 1 week after SPS is well established as evaluated by readouts such as the elevated plus maze (Serova et al., 2013; Souza et al., 2017). The clear stress response in SPS rats after vehicle injection followed by repeated handling confirms this and unfortunately stood in the way of a meaningful comparison of gene expression changes in these animals. The lack of an adrenocortical response of rats in our second experiment, at 30 min after injection, showed that likely the tail incision was the immediate cause of the response in the first experiment.

Our experimental setting was not suited to determine whether negative feedback sensitivity had changed. In our second experiment, the corticosterone treatment mimics the setting in which the enhanced rapid negative feedback was initially observed, but this was defined at the level of adreno corticotropic hormone (ACTH), rather than corticosterone (Liberzon et al., 1997). In other studies, dexamethasone was used, typically 2 h before measuring plasma corticosterone. These studies consistently demonstrate the enhanced suppression of the HPA axis in male rats (Ganon-Elazar and Akirav, 2012; Shafia et al., 2017; Pooley et al., 2018). While the later studies seem to indicate the enhanced genomic effects of glucocorticoids, we do not know whether the SPS-exposed rats in our study actually showed enhanced feedback sensitivity.

The evaluation of gene expression at 30 min after corticosterone could be performed, given the lack of strong injection effects. This showed the pronounced early effects on GR target genes. From a technical point, it is good to note that the strong response to corticosterone occurred not only for upregulated genes but also for the previously established suppressed Drd1a mRNA (Datson et al., 2013; Zalachoras et al., 2013). This argues against an effect on the housekeeping gene used in normalization and for a bona fide difference in responsiveness.

Previous studies have evaluated the expression level of GR in this model. Soon after the development of the model, the increased GR mRNA expression levels were reported in the hippocampus, 1 week after SPS (Liberzon et al., 1999). Also, other studies reported substantially higher (nuclear) GR immunoreactivity in the prefrontal cortex and amygdala 8–15 days after SPS (Knox et al., 2012; Ganon-Elazar and Akirav, 2013; George et al., 2015; Zhang et al., 2020). The data are, however, not immediately intuitive in relation to our previous work, which did not find decreased receptor expression 1 week after SPS (Zhe et al., 2008; Han et al., 2014). However, it is clear from, for example, Cushing’s disease (i.e., mouse models) that there still may be enhanced GR activity despite the homologous downregulation of the GR (Amaya et al., 2021).

Rather than the number of receptors being different, the genomic GR signaling seems to be primed in SPS rats. This notion was previously explored, by looking at GR nuclear translocation 7 days after SPS, and these data suggested enhanced “basal” nuclear GR presence in the amygdala and ventral (but not dorsal) hippocampus based on the Western blot analysis (Moulton et al., 2018). Another study observed high nuclear GR signal in dorsal CA1 and dentate gyrus only in rats that were strongly affected by predator scent exposure (Danan et al., 2021). While the presence of GR nuclear generally follows corticosterone levels, additional regulatory mechanisms are governing nuclear translocation (Mazaira et al., 2021), and these may be relevant to the brain as evidenced by the nuclear GR localization even in adrenalectomized rats (Sarabdjitsingh et al., 2009). FKBP5 is an often studied factor in this respect, i.e., both target and regulator of GR function (Binder, 2009; Matosin et al., 2018; Häusl et al., 2019). In our current data, FKBP5 mRNA levels were affected by SPS in the amygdala but did not explain the enhanced response to corticosterone at 30 min after injection.

The idea that, in PTSD and PTSD models, the GR functionality is changed beyond the negative feedback sensitivity goes back to human studies on lymphocyte GR expression (Yehuda et al., 1991), and, in rodent models, it has logically been extended to higher brain centers, which may be involved in the actual psychopathological symptoms of PTSD (Eagle et al., 2013). Our data add to the notion that GR is involved not only in the initiation of SPS-induced effects (Kohda et al., 2007) but also in their maintenance. The changed GR signaling status might explain why treatment with the GR antagonist RU486/mifepristone can reverse the long-term effects of stressors even when these are administered days to months later in the SPS (Ding et al., 2019, 2020) and other stress paradigms (Loi et al., 2017; Papilloud et al., 2019).

There is still a bias toward research in male experimental animals (Karp and Reavey, 2019). The enhanced negative feedback after SPS seems to be specific to male rats (Pooley et al., 2018). Given that our hypothesis directly derives from the enhanced feedback, the use of male rats makes sense. However, SPS does affect the female rat brain in different ways, and it will be interesting to also test our hypothesis in females in future studies, using the SPS as well as other models of PTSD.

## Conclusion

We observed a strikingly rapid transcriptional response in the hippocampus and amygdala after the administration of corticosterone. It will be interesting to extend these findings from individual cell types (Viho et al., JNE, in revision), functional consequences, and, in the long run, to patients with PTSD.

## Data Availability Statement

The raw data supporting the conclusions of this article will be made available by the authors, without undue reservation.

## Ethics Statement

The animal study was reviewed and approved by China Medical University Animal Care.

## Author Contributions

JD, FH, and OM designed the experiments. JD and XC performed animal experiments. JD and OM performed the statistical analysis and wrote the manuscript. All authors contributed to the article and approved the submitted version.

## Conflict of Interest

The authors declare that the research was conducted in the absence of any commercial or financial relationships that could be construed as a potential conflict of interest.

## Publisher’s Note

All claims expressed in this article are solely those of the authors and do not necessarily represent those of their affiliated organizations, or those of the publisher, the editors and the reviewers. Any product that may be evaluated in this article, or claim that may be made by its manufacturer, is not guaranteed or endorsed by the publisher.
